# High-throughput and simultaneous inertial separation of tumor cells and clusters from malignant effusions using spiral-contraction-expansion channels

**DOI:** 10.1038/s41378-024-00661-0

**Published:** 2024-03-12

**Authors:** Zhixian Zhu, Hui Ren, Dan Wu, Zhonghua Ni, Nan Xiang

**Affiliations:** 1 School of Mechanical Engineering, and Jiangsu Key Laboratory for Design and Manufacture of Micro-Nano Biomedical Instruments, Southeast University, 211189 Nanjing, China; 2 School of Mechanical Technology, Wuxi Institute of Technology, No.1600 Gaolang West Road, 214129 Wuxi, China; 3 Department of Oncology, Jiangyin People’s Hospital, 214400 Jiangyin, China

**Keywords:** Chemistry, Engineering

## Abstract

Tumor cell clusters are regarded as critical factors in cancer pathophysiology, and increasing evidence of their higher treatment resistance and metastasis compared to single tumor cells has been obtained. However, existing cell separation methods that are designed for single tumor cells cannot be used to simultaneously purify tumor cell clusters. To address this problem, we demonstrated a microfluidic approach for the high-throughput, continuous-flow ternary separation of single tumor cells, tumor cell clusters, and WBCs from clinical pleural or abdominal effusions by coupling slanted spiral channels and periodic contraction-expansion arrays. We first systematically explored the influence of particle size and flow rate on particle focusing. The separation performance indicated that 94.0% of WBCs were removed and more than 97% of MDA-MB-231 tumor cells were recovered at a high flow rate of 3500 µL/min. Moreover, more than 90% of tumor cell clusters were effectively preserved after separation. Finally, we successfully applied our device for the ternary separation of single tumor cells, tumor cell clusters, and WBCs from different malignant effusions collected from patients with metastatic cancer. Thus, our spiral-contraction-expansion device has potential as a sample pretreatment tool for the cytological diagnosis of malignant effusions.

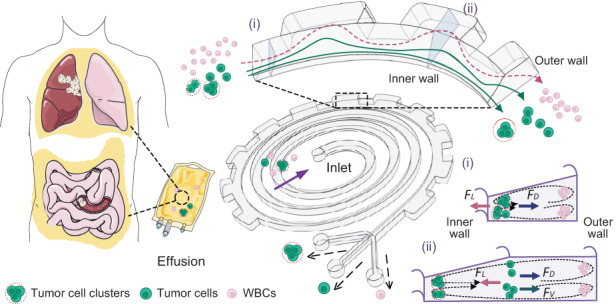

## Introduction

The presence of exfoliated tumor cells and clusters in pleural or abdominal effusions is associated with malignancy, metastatic tropism, or poor prognosis^1–3^. The accurate cytodiagnosis of malignant effusions, particularly the enumeration of tumor cell clusters, holds great clinical importance for cancer diagnosis and prognosis evaluation^4–6^. Tumor cell clusters exhibit 50 times higher metastatic potential than individual tumor cells^7,8^. Immune-affinity-based cell sorting methods have been successfully employed for the separation and detection of individual tumor cells^9–11^, but these approaches suffer from low efficiency in the further purification of tumor cell clusters. Additionally, these immune affinity-based methods cannot obtain live tumor cells and tumor cell clusters for downstream analysis, such as primary culture, drug resistance testing, and gene sequencing^12^.

In addition to immune-affinity-based cell sorting, label-free microfluidics has been employed for the high-throughput enrichment of tumor cells or tumor cell clusters in recent years^13–15^. Intensive research on separating single tumor cells by utilizing the physical properties of tumor cells, such as density^16,17^, size^18–23^, and deformability^24–27^, has achieved great success. With the introduction of external fields, tumor cells can also be separated from background cells based on differences in their dielectric properties^28–30^, magnetic susceptibility^31,32^, or refractive indices^33,34^. Initially, little attention was given to the separation of tumor cell clusters, but recent advances in the understanding of the biogenesis and dissemination of tumor cell clusters have repositioned the separation and identification of tumor cell clusters as critical tasks for preoperative cancer diagnosis^35,36^.

An ideal strategy is to optimize the previous microfluidic sorters designed for separating individual tumor cells to achieve the separation of tumor cell clusters. In earlier research, Sarioglu et al.^37^ developed a Cluster-Chip to independently capture tumor cell clusters using specialized bifurcating traps at a relatively low throughput of 2.5 mL/h. However, additional release processes cause secondary damage to the captured tumor cell clusters. To address this limitation, Au et al.^8^ developed an integrated two-stage array consisting of cylindrical and asymmetric micropillars to isolate viable tumor cell clusters from blood. Moreover, Zeinali et al.^38^ presented a Labyrinth device for the label-free, size-based inertial separation of tumor cells and clusters from metastatic lung cancer patient blood samples. However, the further purification of tumor cell clusters from single tumor cells remained challenging due to the rarity of tumor cell clusters. Edd et al.^39^ reported a nonequilibrium inertial separation array to first remove abundant blood cells and then concentrate tumor cell clusters in a second rerunning step. Despite significant progress, there is still an urgent need for new techniques for the high-throughput, continuous-flow ternary separation of single tumor cells, cell clusters, and blood cells using a single chip.

In this work, we developed a novel spiral-contraction-expansion device by coupling slanted spiral channels with periodic contraction-expansion arrays for high-throughput, continuous-flow ternary separation of tumor cells and tumor cell clusters from a background of blood cells. With the introduction of periodic contraction-expansion arrays, our spiral-contraction-expansion device enabled the size-based ternary separation of cells under the combined action of the inertial lift force, Dean drag force, and local vortex-induced lift force. We first characterized the ternary inertial focusing of differently sized particles in our spiral-contraction-expansion device and optimized the operational flow rate. The separation performance of our device was subsequently evaluated, and the recovery ratio and purity of the tumor cells and clusters were demonstrated. Additionally, we investigated the ability of our device to perform ternary separation of exfoliated tumor cells, tumor cell clusters, and WBCs from clinical pleural or abdominal effusions obtained from cancer patients. Our spiral-contraction-expansion device offers numerous advantages, including label-free and external field-free operation, high-throughput and low-loss separation, and continuous processing in a single step.

## Materials and methods

### Conceptual design and operational principle

**Fig. 1:**
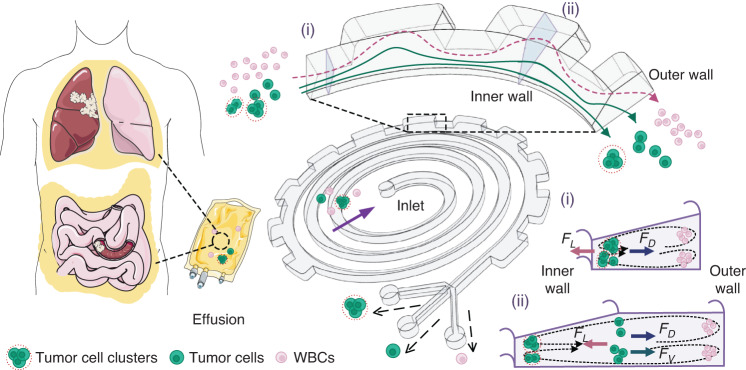
Schematic illustration of high-throughput, continuous-flow ternary separation of tumor cells and clusters from WBCs in large volumes of pleural or abdominal effusions using our spiral-contraction-expansion device. The spiral-contraction-expansion channels include slanted spiral channels with (i) a trapezoidal cross-section and (ii) periodic expansion structures designed at the outer wall

Figure 1 illustrates the conceptual design and working principle of our spiral-contraction-expansion device, which enables the high-throughput, continuous-flow ternary separation of exfoliated tumor cells and clusters from a massive population of blood cells in malignant effusions. Clinical effusion samples collected from cancer patients were simply diluted and then injected into the inlet of our device at a specific flow rate. During the flow through slanted spiral channels, the inertial lift force (*F*_*L*_) induced by shear flow moves large cells toward the inner wall, while the Dean drag force (*F*_*D*_) induced by the curvature of spiral channels pushes small cells toward the outer wall^18,40^. Under the influence of *F*_*L*_ and *F*_*D*_, large tumor cells are focused into a cell string near the inner wall under the dominant effect of *F*_*L*_, whereas small white blood cells (WBCs) gradually migrate to the outer wall and form a band under the dominant effect of *F*_*D*_ (section (i) in Fig. 1). Although blood cells were efficiently removed from the effusion samples, the rare tumor cell clusters remained mixed with single tumor cells due to the binary separation ability of the slanted spiral channels.

To further separate tumor cell clusters from single tumor cells, periodic expansion structures were designed at the outer wall of the last loop of spiral channels, introducing an additional local vortex-induced lift force (*F*_*V*_) toward the outer wall. The spiral-contraction-expansion device thus enabled the high-throughput, continuous-flow ternary separation of tumor cells, clusters, and WBCs. Specifically, the largest tumor cell clusters were focused at a position along the inner wall and single tumor cells near the center of the channel cross-section under the balance of *F*_*L*_, *F*_*D*_, and *F*_*V*_ (section (ii)). Moreover, blood cells were still trapped in the Dean vortex near the outer wall.

### Device design and fabrication

Our spiral-contraction-expansion device was fabricated using a novel chip-on-film technique that involved UV laser cutting, spiral channel assembly, and slanted wall packaging. The detailed fabrication process can be found in our previous work^21^. Specifically, we utilized an ultraviolet laser cutting system (TH-UV200A, Tianhong Laser) to cut four polymer films in predesigned patterns. The short inner wall of the spiral channels was fabricated from 70 µm polymer film, while the high outer wall was cut from a 160 µm film. These two patterned films were then assembled on the same base plate to form open slanted spiral channels. After bonding with the cover plate, our polymer microfluidic device consists of a 4-loop slanted spiral channel and 28 periodic expansion structures along the outer wall (Fig. S1). The cross-section of the slanted spiral channel is trapezoidal, with an inner-wall height of 70 µm, an outer-wall height of 160 µm, and a channel width of 500 µm. The expansion structures are spaced 5 degrees apart along the circumference and have a width of 500 µm. The detailed geometric parameters of our microfluidic device are given in Table S1. The detailed steps for optimizing the device structure can be found in the Supplementary Information (Fig. S2).

### Sample preparation

Four types of standard polystyrene microparticles with diameters of 10, 15, 20, and 25 µm (Thermo Fisher Scientific) were used to characterize the focusing performance before application to cells from clinical effusions. The particle suspensions were diluted with phosphate-buffered saline (PBS, 0.01 M; Sigma‒Aldrich) mixed with 1% Pluronic F-127 (Sigma‒Aldrich) to a concentration of 10^5^ particles/mL.

Human breast tumor cells (MDA-MB-231 cells) obtained from Zhongda Hospital were also used to characterize the separation performance of our device. MDA-MB-231 cells were cultured in an incubator (Forma 381, Thermo Fisher Scientific) at 37 °C in a 5% (v/v) CO_2_ atmosphere using high-glucose Dulbecco’s modified Eagle’s medium (DMEM, Thermo Fisher Scientific) supplemented with 10% fetal bovine serum (FBS, Thermo Fisher Scientific) and 1% penicillin‒streptomycin (Thermo Fisher Scientific). Tumor cell clusters were obtained by dissociating adherent tumor cells from culture flasks using 0.05% trypsin-EDTA solution (Thermo Fisher Scientific). Then, the cell suspension was centrifuged at low speeds, and the cell pellet was gently resuspended in PBS. To prepare the WBC samples, human blood donated by healthy volunteers was lysed using ammonium chloride potassium lysing buffer (Thermo Fisher Scientific) and then diluted to specific concentrations with PBS. Two sets of cell suspensions were mixed to prepare test samples containing a few hundred (154 ± 5 clusters) cell clusters per experiment. The number of cells in our experiments was approximately 10^5^ cells/mL for WBCs and 10^3^ cells/mL for MDA-MB-231 cells.

Clinical pleural or abdominal effusions were collected from 6 patients with metastatic tumors. To eliminate the effects of the viscoelasticity of clinical effusions, each pleural or abdominal effusion sample with a volume of 20 mL was centrifuged at 1000 rpm for 5 minutes, and the pelleted cells were resuspended in the original volume of PBS before separation.

### Immunofluorescence staining

After separation, the cells collected from all outlets were labeled using immunofluorescence staining to identify tumor cells and WBCs. Specifically, cells were first obtained by the centrifugation of collected samples and coated onto Polysine adhesion slides (P4981, Thermo Fisher Scientific) at room temperature. The adherent cells were then fixed with a −20 °C methanol solution for 5 min. After incubation in PBS buffer with 10% normal goat serum (Abcam), the tumor cells were stained with fluorescein isothiocyanate (FITC)-conjugated Pan-CK monoclonal antibody (Thermo Fisher Scientific), while the WBCs were stained with allophycocyanin (APC)-conjugated CD45 antibody (BioLegend) at 4 °C overnight. The fluorescently labeled cells were mounted using Prolong Gold Antifade Mountant with DAPI (Sigma‒Aldrich). Cells that were positive for Pan-CK and DAPI but negative for CD45 were identified as tumor cells, while cells that were positive for CD45 and DAPI but negative for Pan-CK were identified as WBCs.

### Experimental setup and data analysis

The fabricated spiral-contraction-expansion device was clamped within a transparent custom fixture. A high-speed CCD camera (Retiga EXi, QImaging) was mounted on the microscope (IX71, Olympus) to capture the trajectories of the particles and cells through the spiral-contraction-expansion channel. The software ImageJ (National Institutes of Health) was used for vertical stacking of the high-speed images captured during a certain time period. A precise syringe pump (Legato 270, KD Scientific) was used to inject the prepared samples into a spiral-contraction-expansion device at specific flow rates. The cell concentrations were measured by using an automated cell counter (Countess II FL, Thermo Fisher Scientific).

## Results and discussion

### Particle focusing in spiral-contraction-expansion device

**Fig. 2:**
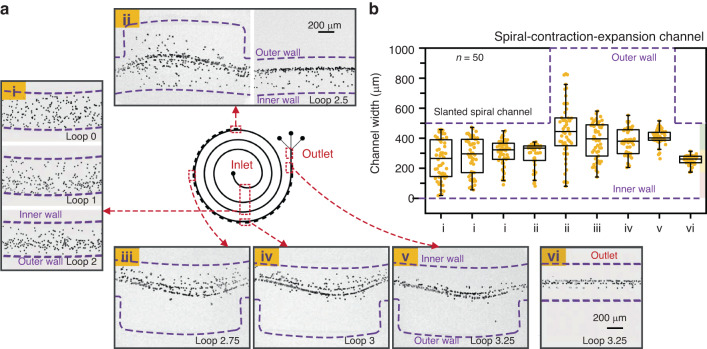
Particle focusing in spiral-contraction-expansion device. **a** Focusing maps of 15 µm particles flowing through the slanted spiral channel (i–ii) and spiral-contraction-expansion channel (ii–vi). **b** Distributions of 15 µm particles at representative locations

Before cell separation, the focusing of polystyrene particles at representative locations was explored to illuminate the separation mechanisms in our spiral-contraction-expansion device. Figure 2a shows the focusing maps of 15 µm particles when flowing through the slanted spiral channel (i-ii) and the spiral-contraction-expansion channel (ii-v) at a flow rate of 3500 µL/min. In the slanted spiral channel (i), the infused particles were randomly dispensed near the inlet. The particle distributions in Fig. 2b indicate that 15 µm particles gradually migrated to the outer wall at loop 3. When flowing into the first spiral-contraction-expansion location (ii), the particle string further migrated to the outer wall with the assistance of the sudden expansion structure. However, the particle string was then squeezed toward the channel centerline by the sudden entry into the contraction structure. When migrating to the 9th spiral-contraction-expansion array (iii), the majority of the particles were focused near the channel centerline. After passing through the whole spiral-contraction-expansion channel (iv and v), 15 µm particles were ultimately focused into a narrow band at the channel centerline (vi). As indicated by the above observations, our spiral-contraction-expansion device was able to enhance the focusing effect and modify the focusing position by combining slanted spiral channels with periodic contraction-expansion arrays, enabling ternary particle separation.

### Ternary inertial focusing of differently sized particles

**Fig. 3:**
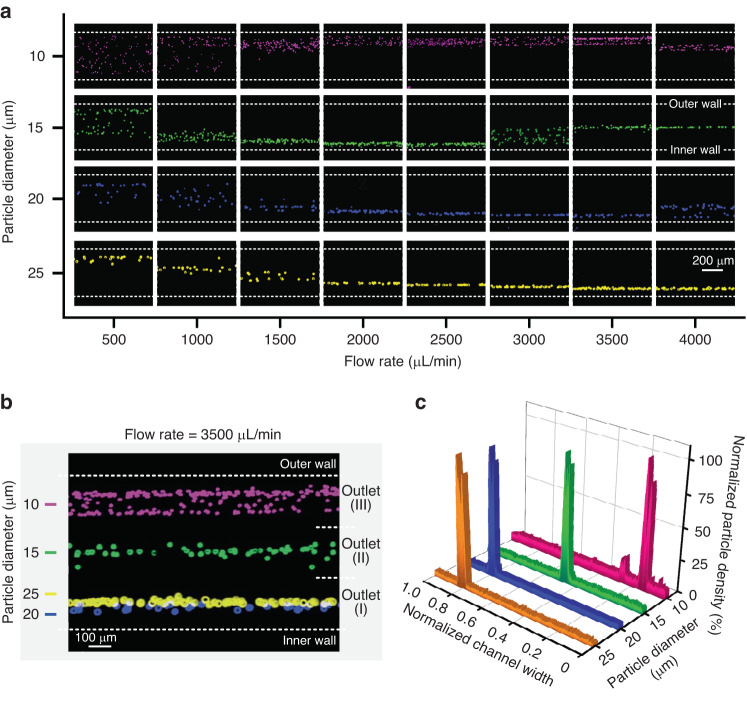
Ternary inertial focusing of differently sized particles. **a** Focusing maps of 10, 15, 20, and 25 µm particles under flow rates of 500–4000 µL/min with intervals of 500 µL/min in the spiral-contraction-expansion device. **b** Ternary focusing of particles at the optimal flow rate of 3500 µL/min near the channel outlet. **c** Normalized density profile of four differently sized particles at a flow rate of 3500 µL/min across the channel width

The ternary focusing performance of the spiral-contraction-expansion device for the size-based separation of different particles was then tested. A series of particles with diameters ranging from 10 to 25 µm were selected to simulate WBCs (10 µm), single tumor cells (15 µm), and tumor cell clusters (20-25 µm)^41–43^. To evaluate the evolution of ternary focusing with respect to particle size and flow rate, focusing maps of differently sized particles near the channel outlet were obtained and visualized (Fig. 3a). At flow rates of 500-1000 µL/min, 10 µm particles were randomly distributed across the channel width. At flow rates of 1500 and 3000 µL/min, only 10 µm particles were pushed to the outer wall by the dominant *F*_*D*_, while 15-25 µm particles were focused into a string along the inner wall by the dominant *F*_*L*_, revealing the possibility of binary separation of blood cells and tumor cells. When the flow rate was increased to 3500–4000 µL/min, the local vortexes generated from the periodic expansion structures forced the 15 µm particles to a new equilibrium position at the channel centerline. Particles with diameters of 20 µm and 25 µm occupied the focusing positions near the inner wall due to the higher *F*_*L*_ at the same flow rate. As expected, ternary focusing of differently sized particles was realized by coupling the slanted spiral channels with periodic expansion structures to introduce competition among *F*_*D*_, *F*_*L*_, and *F*_*V*_. Figure 3b, c illustrates that particles with four different diameters occupied three different focusing positions across the channel width under an optimal flow rate of 3500 µL/min. Specifically, 10 µm particles were focused into a cell belt 373–456 µm away from the inner wall. A single focusing train of 15 µm particles appeared along the channel centerline (~253 µm), while the 20 and 25 µm particles were focused tightly along the inner wall (~83 µm). The normalized density profile of these particles indicated the successful ternary separation of differently sized particles at a high flow rate of 3500 µL/min.

### Characterization of cell separation performance

We next characterized the cell separation performance of our device by using MDA-MB-231 breast tumor cells (containing both single cells and clusters) and WBCs at flow rates ranging from 500–4000 µL/min. Fig. S3a illustrates the dynamic focusing performance of MDA-MB-231 cells in a wide flow rate range of 500–4000 µL/min. After flowing through the spiral-contraction-expansion channel, MDA-MB-231 cells were focused near the inner wall and the channel centerline at flow rates of 3000-4000 µL/min. Moreover, the WBCs migrated to the outer wall for removal at flow rates ranging from 2500-3500 µL/min, as shown in Fig. S4a. To evaluate the cell separation performance of our device, a lysed blood sample (with a WBC concentration of ~10^5^ cells/mL) spiked with MDA-MB-231 cells (~10^3^ cells/mL) was injected into the channel inlet at the optimal flow rate of 3500 µL/min. After counting the cells in the liquid collected from the outlets, 93.4% of the MDA-MB-231 cells were recovered from outlet II, while 4.2% of the MDA-MB-231 cells (cell clusters) were recovered from outlet I. Moreover, 94.0% of the WBCs were simultaneously removed from outlet III at 3500 µL/min (Figs. S3b and S4b). In addition, Fig. S5a shows microscopy images of tumor cells before and after separation, indicating that the separated cells exhibited a high viability of 95.0% (Fig. S5b) and could be recultured within 72 h (Fig. S5c).

To investigate potential damage to cell clusters during high-throughput separation, the numbers of tumor cell clusters were compared before and after separation (Fig. S6). The statistical results obtained from the captured microscopy images showed that the proportion of 2-cell clusters increased slightly from 73% ± 3.5% to 83% ± 3.5%, while that of multiplex cell clusters (cell number ≥ 3) decreased in the range of 1–4%. It was speculated that a fraction of the large clusters broke into smaller 2-cell clusters or single tumor cells due to hydrodynamic effects. However, the vast majority of tumor cell clusters were well preserved. It can be concluded that the spiral-contraction-expansion device effectively preserved the integrity of cell clusters.

### Separation of tumor cells and clusters from clinical effusions

**Fig. 4:**
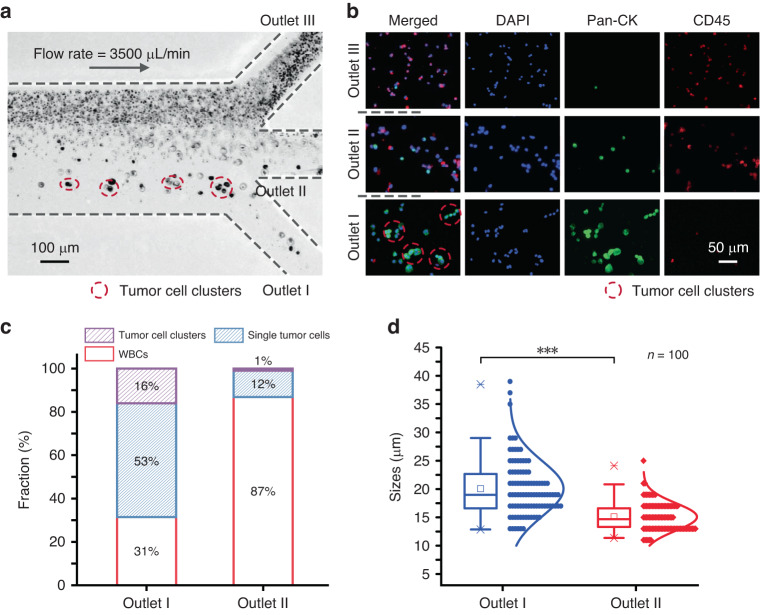
Separation of tumor cells and clusters from clinical effusions. **a** A bright-field image depicting the cell distribution near the outlet bifurcation region of our spiral-contraction-expansion device. **b** Fluorescence microscopy images of the cells isolated from the outlets after immunofluorescence staining. The cells were stained with DAPI (blue), Pan-CK (green), and CD45 (red). **c** Proportions of WBCs, single tumor cells, and tumor cell clusters collected from target outlets I and II. **d** Size distributions of tumor cells (in both single and cluster forms) collected from target outlets I and II (***p value < 0.001)

Then, we employed our spiral-contraction-expansion device for the ternary separation of WBCs, single tumor cells, and tumor cell clusters from clinical pleural effusions. A total of 20 mL of pleural effusion fluid was collected from a patient with advanced breast cancer, pretreated, and pumped into the device at 3500 µL/min. Figure 4a illustrates the cell distribution near the outlets. One waste outlet was used to collect the WBCs, while the two target outlets were used to recover single tumor cells and tumor cell clusters. A row of tumor cell clusters with multiple cells marked with red circles was observed near outlet I. A band of relatively large single tumor cells was focused near the channel centerline and could be exported by the central outlet II. A high density of blood cells was pushed toward the outer wall and removed by outlet III.

The cell suspensions collected from the three outlets were visualized using immunofluorescence staining and counted under a microscope (Fig. 4b). The recovered tumor cells (both single cells and cell clusters) were defined by the Pan-CK^+^ (green)/CD45^−^ (red)/DAPI^+^ (blue) phenotype, while the blood cells were defined by the Pan-CK^−^/CD45^+^/DAPI^+^ phenotype. Merged fluorescence microscopy images revealed that tumor cell clusters were collected from outlet I, while mostly single tumor cells mixed with similar-sized WBCs were collected from outlet II. To quantitatively assess the separation of single tumor cells and clusters, the fraction of cells collected from the two target outlets was calculated, as shown in Fig. 4c. The fraction of tumor cell clusters was increased from less than 1% at the inlet to nearly 16% at outlet I, while the fraction of single tumor cells at outlet II was 12%. These results indicated that our microfluidic device could achieve the high-throughput, continuous-flow recovery of single tumor cells and clusters from two target outlets. The size distributions of the tumor cell clusters (*n* = 100), single tumor cells (*n* = 100), and WBCs were measured and calculated by determining the cell density using ImageJ (Fig. 4d). The average equivalent diameter of the tumor cell clusters recovered from outlet I was 20.1 µm. The maximum equivalent diameter of the recovered tumor cell clusters was 38.5 µm. The equivalent diameter of cells from outlet II was 15.1 µm, with a maximum of 24.1 µm and a minimum of 11.4 µm. These results demonstrated that our spiral-contraction-expansion device could separate single tumor cells and clusters with a wide size distribution between two distinct outlets with excellent efficiency while removing the background WBCs.

### Comparative analysis of tumor cell clusters

**Fig. 5:**
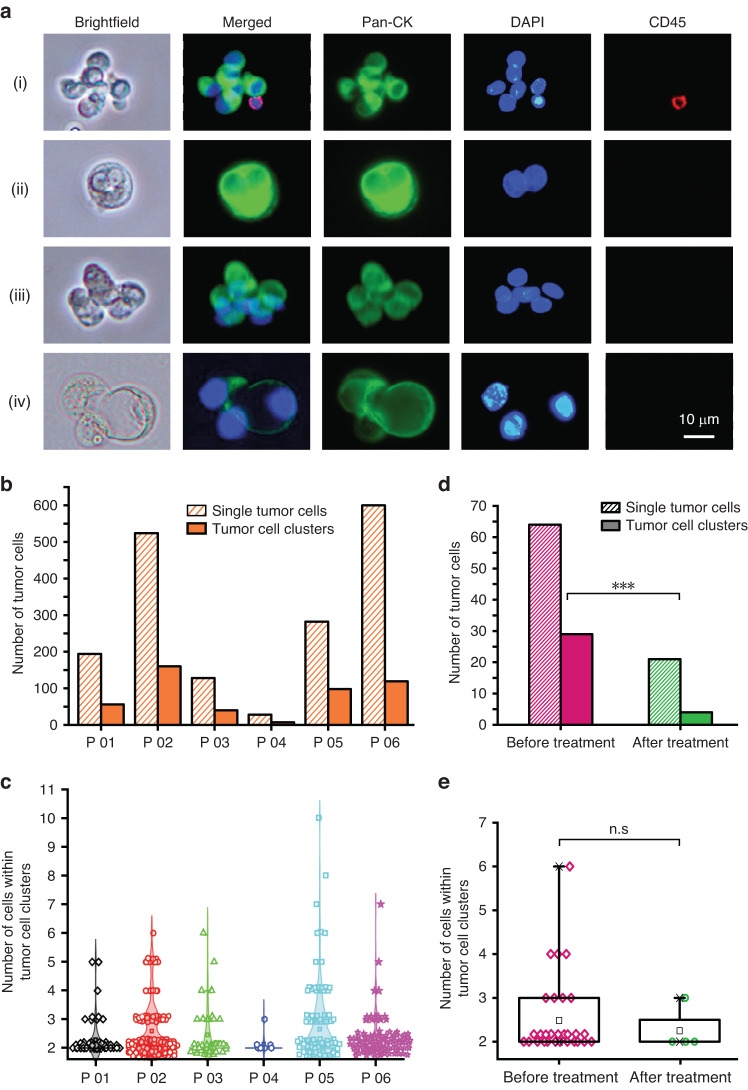
Separation of tumor cells and clusters from cancer patients (*n* = 6) using our spiral-contraction-expansion device. **a** Fluorescence microscopy images illustrating the recovered tumor cell clusters identified by Pan-CK + , DAPI + , and CD45-. **b** The number of single tumor cells and clusters isolated from cancer patient effusions (*n* = 6). **c** Number distribution of cells within the collected tumor cell clusters (*n* = 6). **d** Comparison of isolated tumor cells and clusters from a lung cancer patient before and after treatment (*** indicates a p value less than 0.001). **e** Comparison of the number of cells within tumor cell clusters recovered from a lung cancer patient before and after treatment (n.s indicates a *p* value > 0.05)

We further utilized our device to collect tumor cell clusters from clinical effusions. Typical fluorescence microscopy images of the recovered tumor cell clusters are shown in Fig. 5a. Notably, the cluster in Fig. 5a(i) contained seven cell nuclei, including one WBC with a small red outline stained by DAPI. The clusters in Fig. 5a(ii) contained only two tumor cells. The cluster shown in Fig. 5a(iii) was similar in size to cluster (i), with seven tumor cells in the cluster. The cluster in Fig. 5a(iv) consisted of three cells, one large cell and two smaller cells.

Samples (20 mL) of pleural or abdominal effusion were collected from six patients diagnosed with lymphoma or lung, breast, pancreatic, liver, or ovarian cancer. The characteristics of the malignant effusions are presented in Supplementary Table S2. As shown in Fig. 5b, tumor cell clusters were identified in 6/6 patients. The number of cells within the tumor cell clusters ranged from 2–10 (Fig. 5c). As shown in Fig. 5b and c, the effusion of Patient No. 6 had the highest overall number of single tumor cells and clusters, with 100 of 119 being 2-cell clusters. The number of tumor cells (including single and cluster forms) recovered from the effusion from Patient No. 4 was the lowest, and the majority of the clusters were 2-cell clusters (6 out of 7). The number of clusters recovered from Patient No. 2 was the highest, including 98/160 2-cell clusters, 41/160 3-cell clusters, 9/160 4-cell clusters, and 12/160 ≥ 5-cell clusters.

To compare the numbers of tumor cells and clusters before and after treatment, 20 mL of pleural effusion was initially collected from a patient with stage IV lung cancer and then pumped into our device. After one cycle of regular antitumor treatment, pleural effusions were collected again from this patient, and the collected sample was analyzed, as shown in Fig. 5d-e. A significant decrease in cell quantity was observed after treatment, from 29 to 4 clusters and from 64 to 21 single tumor cells. Additionally, after treatment, the 4-cell clusters (*n* = 3) and 6-cell clusters (*n* = 1) were eliminated, while the number of 2-cell clusters decreased dramatically from 21 to 3. The number of single tumor cells and clusters in the effusions decreased after treatment. The number and size distribution of tumor cell clusters may promptly reflect the disease progression of cancer patients. The above results demonstrate that our spiral-contraction-expansion device holds extensive value in clinical application.

## Conclusion

In this work, a novel combination of slanted spiral channels and periodic contraction-expansion arrays was employed for the inertial separation of tumor cells and clusters from clinical pleural or abdominal effusions. After a systematic exploration of the influence of flow rate on particle focusing, our spiral-contraction-expansion device successfully achieved ternary size-based focusing of 10 µm, 15 µm, and 20-25 µm particles at a high throughput of 3500 µL/min. The separation of the cell samples indicated that 94.0% of the WBCs were removed and that more than 97% of the MDA-MB-231 tumor cells were recovered at 3500 µL/min. Moreover, more than 90% of the tumor cell clusters were effectively preserved after separation. Finally, we demonstrated that our device was capable of the ternary separation of single tumor cells, tumor cell clusters, and WBCs from malignant effusions from cancer patients (*n* = 6). In clinical application, our device increased the fraction of tumor cell clusters from less than 1% (inlet) to 16% (outlet). The tumor cell clusters recovered from a lung cancer patient before and after treatment were compared, and both the cell number within the clusters and the size distribution were significantly reduced after treatment. Our spiral-contraction-expansion channels allow the label-free, continuous-flow, high-throughput separation of tumor cells and clusters from cancer patient effusions in a single step. Therefore, our device is promising for improving diagnosis and treatment assessment by the analysis of malignant effusions in the clinic.

### Supplementary information

Supplemental Material
