# Supplementary material for: High-throughput and simultaneous inertial separation of tumor cells and clusters from malignant effusions using spiral-contraction-expansion channels
Source: Microsyst Nanoeng. 2024 Mar 12;10:36. doi: 10.1038/s41378-024-00661-0 (PMC10933397; doi:10.1038/s41378-024-00661-0)
Supplement: Supplementary file 1 — Supplemental Material [file 41378_2024_661_MOESM1_ESM.docx]

**Supporting information**

**High-throughput and simultaneous inertial separation of tumor cells and clusters from malignant effusions using spiral-contraction-expansion channels**

Zhixian Zhu^1+^, Hui Ren^2+^, Dan Wu^3^, Zhonghua Ni^1^, and Nan Xiang^1^*

^1^School of Mechanical Engineering, and Jiangsu Key Laboratory for Design and Manufacture of Micro-Nano Biomedical Instruments, Southeast University, Nanjing, 211189, China.

^2^School of Mechanical Technology, Wuxi Institute of Technology, No.1600 Gaolang West Road, Wuxi, 214129, China.

^3^Department of Oncology, Jiangyin People’s Hospital, Jiangyin, 214400, China.

^+^ Zhixian Zhu and Hui Ren contributed equally to this work.

***Corresponding author:** Nan Xiang, nan.xiang@seu.edu.cn

**Optimization of device structure**

The effects of expansion width on particle focusing were systematically explored for better understanding of the separation mechanisms in spiral-contraction-expansion channels. The particle focusing performances of channels with various expansion widths of 0-900 µm were first tested using 15 and 10 µm particles across a wide range of flow rates of 500-4000 µL/min. The 15 and 10 µm particles were used to simulate the tumor cells and WBCs, respectively. Fig. S2a shows the focusing maps illustrating the distributions of 15 and 10 µm particles when flowing through individual slanted spiral channels. At the flow rates of 2500 and 3000 µL/min, 15 µm particles focused into a thin string near the inner wall, while 10 µm particles migrated toward the outer wall. Upon introducing the periodic expansion structures, 15 µm particles migrated and focused near the channel centerline at a flow rate of 3500 µL/min (Fig. S2b). Compared with the focusing position of 10 µm particles, the optimum width of periodic expansion structures was chosen to be 500 µm, achieving a clear binary focusing of 15 and 10 µm particles. At the channel outlets, most of 10 µm particles were removed from outlet III, while 15 µm particles were perfectly focused along the channel centerline and recovered from outlet II.


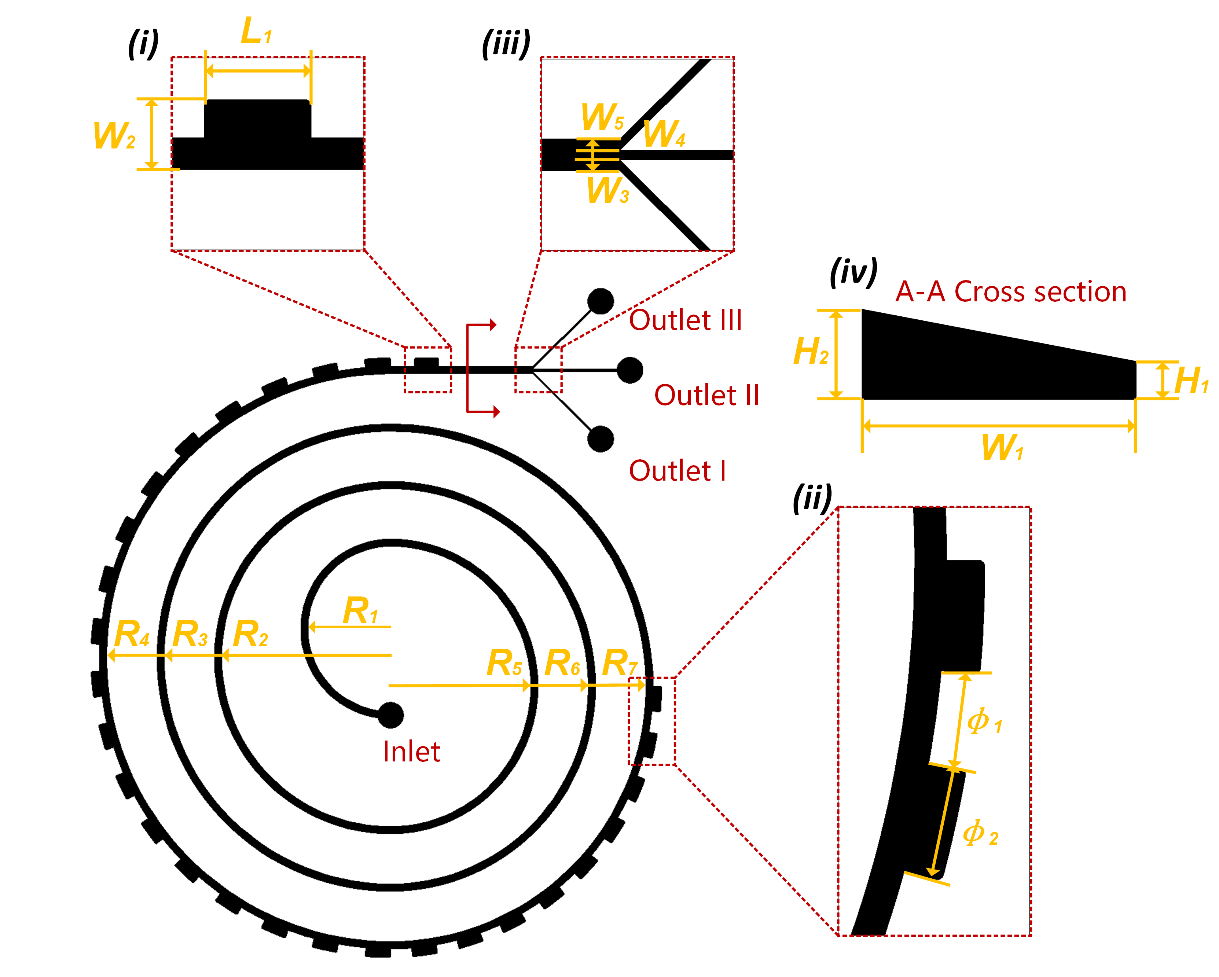


**Fig. S1.** CAD drawing illustrating the detailed structures of our spiral-contraction-expansion channel.

**
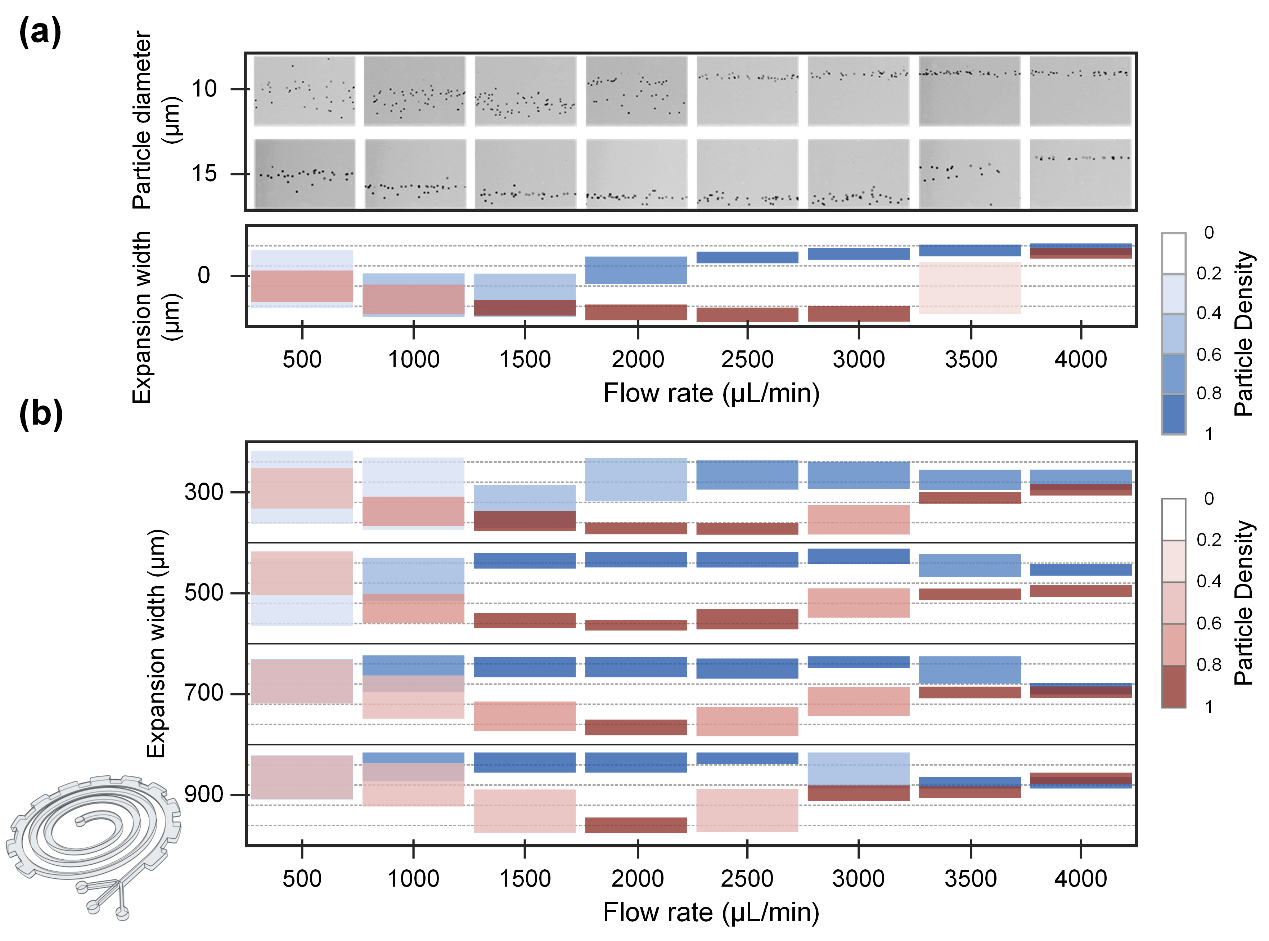
**

**Fig. S2.** Focusing performances of 10 and 15 µm particles in spiral-contraction-expansion channels under various flow rates ranging from 500 to 4000 µL/min. (a) Composite images and the corresponding density distribution diagrams illustrating the distributions of 10 and 15 µm particles across the width of channels without expansion structures. (b) Density distribution diagrams of 10 and 15 µm particles in channels with different expansion widths ranging from 0 µm to 900 µm with an interval of 200 µm.

**
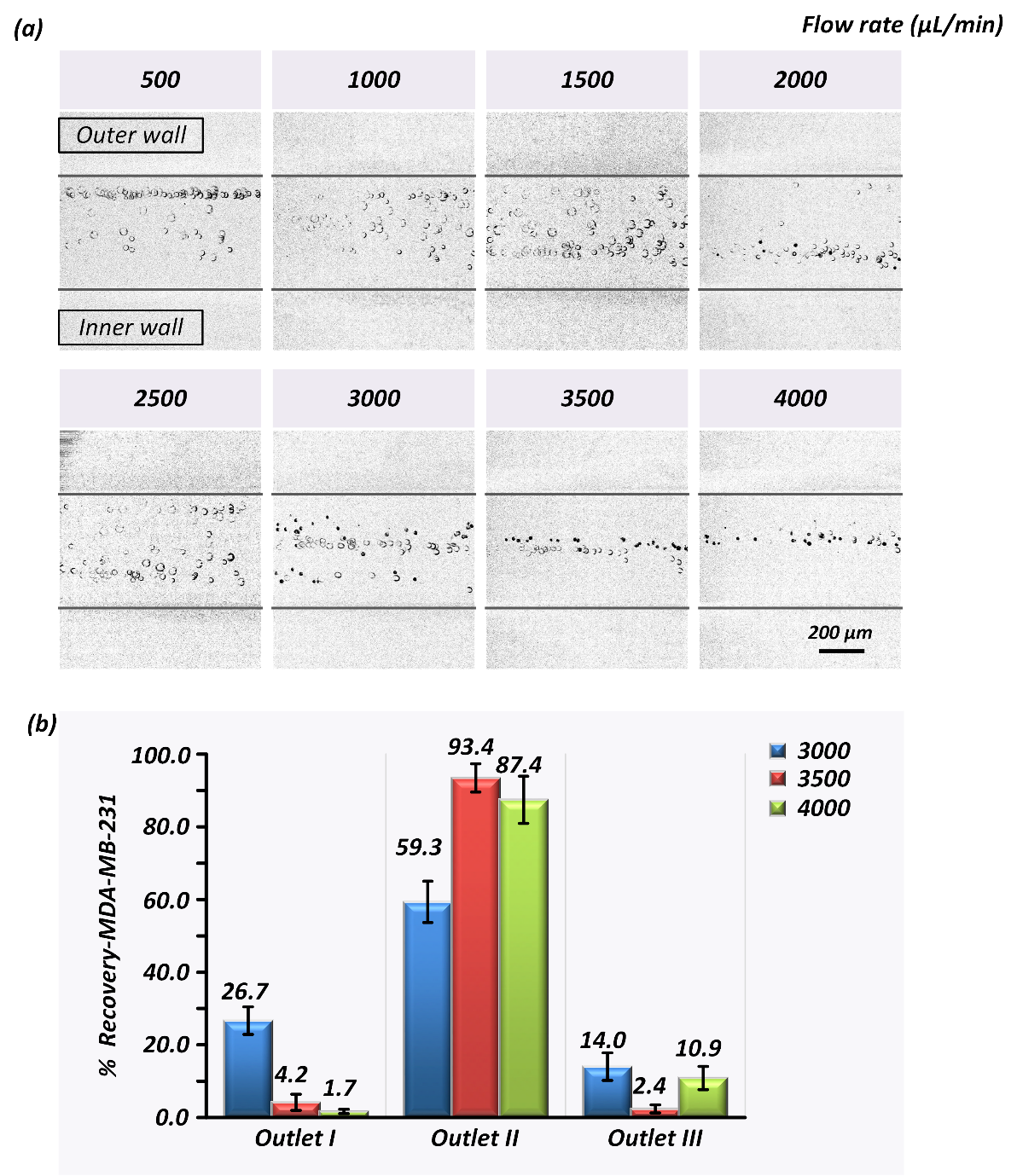
**

**Fig. S3.** Cell separation performance of our device at various flow rates. (a) Stacked bright-field images of MDA-MB-231 tumor cells illustrating the cell focusing positions under a wide flow rate range of 500-4000 µL/min. (b) Recovery efficiencies of MDA-MB-231 tumor cells from three outlets (Outlets I, II, and III) at the flow rates of 3000 µL/min, 3500 µL/min, and 4000 µL/min.

**
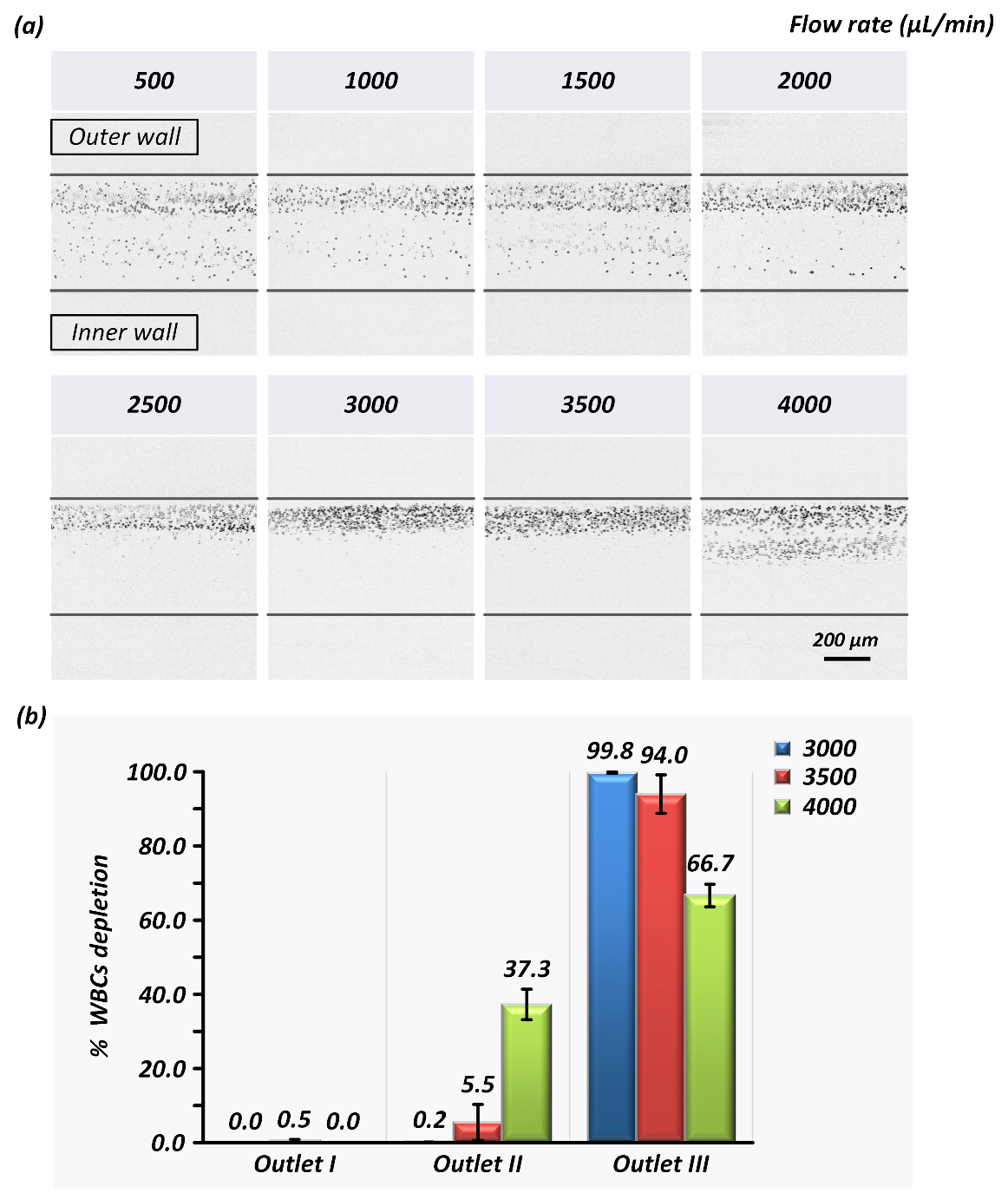
**

**Fig. S4.** Cell separation performance of our device at various flow rates. (a) Stacked bright-field images of WBCs illustrating the cell focusing positions under a wide flow rate range of 500-4000 µL/min. (b) Depletion rate of WBCs from three outlets (Outlet I, II, and III) at typical flow rates of 3000 µL/min, 3500 µL/min, and 4000 µL/min.


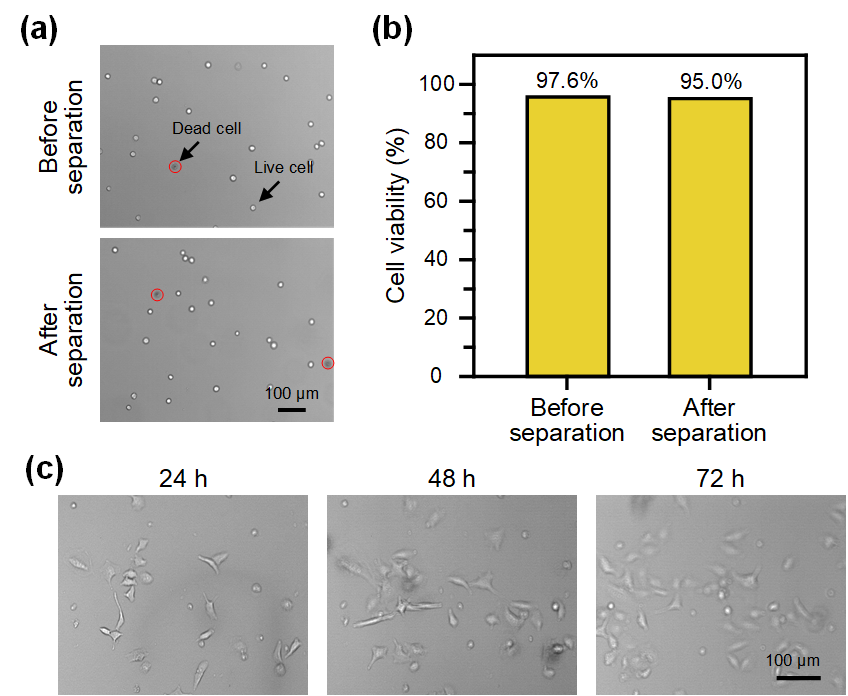


**Fig. S5.** (a) Bright field images of trypan blue staining before and after cell separation. (b) Changes in cell viability before and after separation. (c) Microscopic images of the separated tumor cells re-cultured for 24, 48, and 72 hours.

**
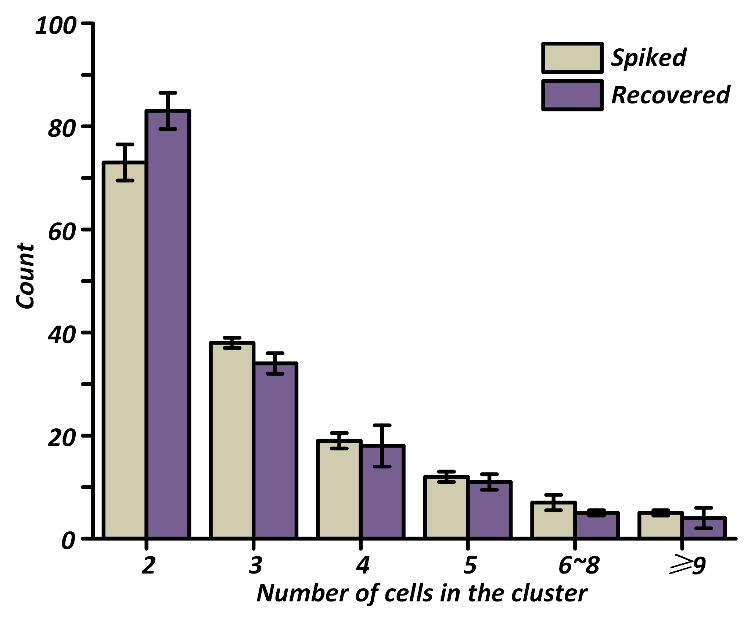
**

**Fig. S6.** Numbers of various tumor cell clusters before and after separation.

**Table S1.** Detailed geometric parameters.

| **Parameter** | **(mm)** | **Parameter** | **(mm)** |
| --- | --- | --- | --- |
| **R_1_** | 5.75 | **H_1_** | 0.07 |
| **R_2_** | 11.75 | **H_2_** | 0.16 |
| **R_3_** | 15.75 | **W_1_** | 0.5 |
| **R_4_** | 19.75 | **L_1_** | 1.67 |
| **R_5_** | 9.75 | **W_2_** | 1.1 |
| **R_6_** | 13.75 | **W_3_** | 0.17 |
| **R_7_** | 17.75 | **W_4_** | 0.15 |
| **Φ_1_** | 5° | **W_5_** | 0.18 |
| **Φ_2_** | 5° | **Expansion array** | N=28 |

**Table S2.** Patient characteristics (n=6).

| **Patient ID** | **Type of effusion** | **Gender** | **Age** | **Diagnosis** | **Cancer stage** |
| --- | --- | --- | --- | --- | --- |
| 1 | Pleural effusion | Female | 64 | Lung cancer | IV |
| 2 | Ascites | Female | 61 | Breast cancer |  |
| 3 | Pleural effusion | Male | 58 | Pancreatic cancer | IV |
| 4 | Ascites | Male | 57 | Liver cancer | IV |
| 5 | Ascites | Female | 75 | Ovarian cancer |  |
| 6 | Pleural effusion | Female | 80 | Lymphoma |  |
